# An exploration of lifestyle beliefs and lifestyle behaviour following stroke: findings from a focus group study of patients and family members

**DOI:** 10.1186/1471-2296-11-97

**Published:** 2010-12-08

**Authors:** Maggie Lawrence, Susan Kerr, Hazel Watson, Gillian Paton, Graham Ellis

**Affiliations:** 1 School of Health/Institute for Applied Health Research, Glasgow Caledonian University, Glasgow, G4 0BA, Scotland, UK; 2 School of Health, Glasgow Caledonian University, Glasgow, G4 0BA, Scotland, UK; 3 NHS Greater Glasgow and Clyde, Royal Alexandra Hospital, Paisley, PA2 9PN, Scotland, UK; 4 NHS Lanarkshire, Medicine for Elderly, Monklands Hospital, Airdrie, ML6 0JS, Scotland, UK

## Abstract

**Background:**

Stroke is a major cause of disability and family disruption and carries a high risk of recurrence. Lifestyle factors that increase the risk of recurrence include smoking, unhealthy diet, excessive alcohol consumption and physical inactivity. Guidelines recommend that secondary prevention interventions, which include the active provision of lifestyle information, should be initiated in hospital, and continued by community-based healthcare professionals (HCPs) following discharge. However, stroke patients report receiving little/no lifestyle information.

There is a limited evidence-base to guide the development and delivery of effective secondary prevention lifestyle interventions in the stroke field. This study, which was underpinned by the Theory of Planned Behaviour, sought to explore the beliefs and perceptions of patients and family members regarding the provision of lifestyle information following stroke. We also explored the influence of beliefs and attitudes on behaviour. We believe that an understanding of these issues is required to inform the content and delivery of effective secondary prevention lifestyle interventions.

**Methods:**

We used purposive sampling to recruit participants through voluntary sector organizations (29 patients, including 7 with aphasia; 20 family members). Using focus group methods, data were collected in four regions of Scotland (8 group discussions) and were analysed thematically.

**Results:**

Although many participants initially reported receiving no lifestyle information, further exploration revealed that most had received written information. However, it was often provided when people were not receptive, there was no verbal reinforcement, and family members were rarely involved, even when the patient had aphasia. Participants believed that information and advice regarding healthy lifestyle behaviour was often confusing and contradictory and that this influenced their behavioural intentions. Family members and peers exerted both positive and negative influences on behavioural patterns. The influence of HCPs was rarely mentioned. Participants' sense of control over lifestyle issues was influenced by the effects of stroke (e.g. depression, reduced mobility) and access to appropriate resources.

**Conclusions:**

For secondary prevention interventions to be effective, HCPs must understand psychological processes and influences, and use appropriate behaviour change theories to inform their content and delivery. Primary care professionals have a key role to play in the delivery of lifestyle interventions.

## Background

Stroke is a major cause of mortality, disability and family disruption [1-3]. It also has a significant economic impact in terms of acute intervention and long-term health and social care [4]. Globally, despite the many advances in prevention and treatment of stroke, the absolute number of strokes continues to rise due to the ageing demographic of the population [5]. Following stroke, patients are at risk of recurrent stroke (approximately 25% within five years) and other vascular events [6,7]. Recurrent stroke may result in death (25% within 28 days), or increased risk of further disability, dependence and institutionalisation [6,7]. Risk factors include lifestyle behaviours, e.g. tobacco use, unhealthy diet, excessive alcohol consumption and physical inactivity. Evidence-based guidelines recommend that secondary prevention interventions should be initiated while the patient is in hospital, and continued by healthcare professionals (HCPs) working in primary care settings [8,9]. Interventions should be multimodal, i.e. they should include the prescription of secondary prevention medication (e.g. antihypertensives, statins) in conjunction with the active provision of lifestyle information, and education regarding behaviour modification strategies [10].

Lifestyle information delivered as an element of a secondary prevention intervention can help people to instigate and maintain lifestyle change(s). Such changes may save lives and reduce the extension of disability, thus diminishing disruption to individuals and their families, and also the economic burden for public services [8,11,12]. However, a recent survey found that almost 50% of stroke patients reported receiving no dietary advice, and one third reported receiving no information about physical activity [13]. In another study, 54% of stroke patients reported receiving no lifestyle information at all [14]. These findings indicate that there is a need for improved management of lifestyle risk factors, beginning with the provision of information. To date, little research has been conducted in this area. A scoping search undertaken as the first stage of a systematic review of the literature [15,16] identified only two randomised controlled trials (RCTs) that had evaluated behavioural lifestyle interventions in stroke populations [17,18]. The lifestyle issues addressed in these multimodal interventions included tobacco use, diet, alcohol consumption and physical activity. Although one of the studies demonstrated a significant positive effect on changes to diet and physical activity at 12-month follow-up [18], neither of the two RCTs effected sustained positive behaviour change across the range of lifestyle risk factors [17,18]. Although it is known that families exert considerable influence on lifestyle behaviour [19], none of the studies retrieved in the scoping exercise had adopted a family-centred approach to the delivery of secondary prevention interventions. It was also noted that none of the interventions appear to have addressed the complex nature of behaviour change using appropriate psychological theories. Consequently, we hypothesised that such interventions are more likely to be effective if they are informed by an appropriate theoretical approach and if they take into account the influence of family on lifestyle behaviour.

Therefore, acknowledging the need for an effective intervention designed to reduce recurrent stroke risk, and recognising the limitations of previous work in this area, we used the Medical Research Council's framework for the development and evaluation of complex interventions [20], to guide the development of a structured programme of family-centred secondary prevention research [16,21]. Importantly, the programme is underpinned by theories [22] that facilitate understanding of the mechanisms that influence lifestyle behaviour change, or lack of behaviour change, following stroke [23].

**Figure 1:**
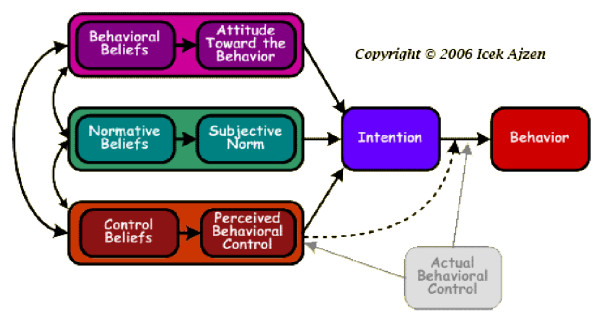
**Diagrammatic representation of the Theory of Planned Behaviour**.

Two complementary theories were selected to underpin our programme of research, a family systems theory [24] and the Theory of Planned Behaviour (TPB) [25]. TPB is of particular relevance to this study as it describes and explains behaviour/behaviour change as determined by intentions to engage/not to engage in specific behaviours e.g. smoking. Intentions are informed by attitudes, motivation and perceived behavioural control; factors embedded in/influenced by intersubjective relationships within the family (Figure 1). In order to develop and deliver effective secondary prevention behavioural interventions, HCPs need to be aware of and understand the beliefs and attitudes of patients and their families, and their needs regarding secondary prevention lifestyle information. This evidence is lacking in the stroke literature. Therefore, we undertook the qualitative study reported here, which aimed to explore the beliefs and behaviours of patients and their families following stroke.

## Methods

### Study design, participants and data collection

We held focus groups with people aged ≥18 who had had a stroke and were living at home, and with family members of adults who had had a stroke. Focus groups were used to collect data as they encourage interaction amongst participants, highlight areas of agreement or disagreement within a group, and enable observation of non-verbal communication [26,27]. Focus groups have been used successfully with people who have had a stroke, including those with communication impairments [28].

Using purposive sampling methods, we recruited participants through voluntary sector organisations (VSOs) from four regions in Scotland, which included urban and rural populations, in deprived and affluent areas. Participants who had had a stroke had had their stroke at least six months previously. Adults with aphasia were actively recruited to ensure that their views were ascertained [29]. Recruitment packs included information sheets and consent forms in either standard print format or in an easy-access format i.e. information in a format accessible by people with aphasia [30-32], (see additional files 1 and 2). Prior to the commencement of recruitment and data collection, ethical approval was obtained from Glasgow Caledonian University's Ethics Committee.

The focus groups were held in the VSOs' usual meeting places. Seven of the focus groups were digitally recorded and transcribed, and detailed field notes were made for the first focus group (FG1), which was not recorded, due to equipment failure. Each focus group lasted approximately 90 minutes. The groups were facilitated by an experienced focus group moderator (ML or SK) and a co-moderator (SK or RW (see acknowledgements)); communication support was provided by GP, a Speech and Language Therapist. A semi-structured topic guide was developed as a basis for the focus group discussions. The topic guide for people who had had a stroke is provided in additional file 3.

### Data analysis

The focus group data were analysed thematically using a framework approach [33], which, moving from the inductive to the deductive, reflected the accounts and descriptions of the participants within the precepts of the theoretical framework i.e. the TPB. QSR NVIVO v.7 (qualitative analysis software) was used to support this process. In the first stage, ML immersed herself in the data, by listening repeatedly to the interview recordings, often whilst simultaneously reading the transcripts, to gain an impression of the data as a whole. During the second stage, ML identified meaning units and subsequently, from the convergence of meaning units, identified themes. The themes were then discussed and agreed with SK, and considered in the light of the TPB. In the third stage, ML, SK and HW reviewed the themes, checked them against the data, and further refined them. The themes were finally described in terms of the principal tenets of the TPB i.e. Information, Behavioural beliefs, Normative beliefs and Control beliefs [25]. TPB was employed to facilitate an interpretation and description of the findings, rather than to predetermine the nature of the data. Verbatim quotes from the focus groups are used to illustrate the findings.

## Results

Eight focus groups were conducted between March and July 2008. Twenty family members (FM) and 29 people who had had a stroke (PwS), including seven people with aphasia (PwA) participated in the focus groups. Each group had between two and 13 participants; the average number of participants was six. We used purposive sampling to facilitate the recruitment of a sample reflective of a range of demographic characteristics. Predominantly, the participants were members of support groups convened in less affluent/deprived areas, which reflects the socio-economic profile associated with stroke incidence [5]. The composition of the groups was mixed and was determined pragmatically i.e. according to the pre-existing profile of the support group's membership. Three groups comprised only people who had had a stroke (there was one person with aphasia in each of these groups), and three groups comprised only family members. Two groups comprised people who had had a stroke and family members, both of these groups included people with post-stroke aphasia. Demographic data are presented in Tables 1 and 2.

**Table 1:** Demographic details: people who had had a stroke

Variable	Frequency (%)
**Sex**	
male	16 (55.2)
female	13 (44.8)

**Age**	

35-44 yrs	3 (11.1)
45-54 yrs	7 (25.9)
55-64 yrs	4 (14.8)
65-74 yrs	6 (22.2)
75-84 yrs	7 (25.9)

Median 62 yrs; range 37-81 yrs	

**Marital status**	
single	3 (10.3)
married	16 (55.2)
divorced	3 (10.3)
widowed	7 (24.1)

**Living arrangements**	
alone	10 (34.5)
with other/s	19 (65.5)

**Employment**	
paid employment	3 (10.3)
unemployed	6 (20.7)
retired	19 (65.5)
voluntary work	1 (3.4)

**Previous stroke**	
yes	9 (31)
no	20 (69)

**Table 2:** Demographic details: family members

Variable	Frequency %
**Sex**	
male	11 (55)
female	9 (45)

**Age**	
35-44 yrs	1 (5)
45-54 yrs	3 (15)
55-64 yrs	6 (30)
65-74 yrs	7 (35)
75-84 yrs	3 (15)

Median 64 yrs; range 42-79 yrs	

**Marital status**	
single	3 (15)
married	16 (80)
divorced	1 (5)

**Living with PwS/PwA**	
yes	17 (85)
no	3 (15)

**Employment**	
paid employment	3 (15)
unemployed	3 (15)
retired	14 (70)

**Caring input**	
cooking	15 (75)
shopping	17 (89.5)

Key: PwS: person who has had a stroke; PwA: person who has aphasia following stroke

### Information

Information is a central component of the TPB in the form of behaviour relevant beliefs. Information, whether it is correct or incorrect, works in favour of, or against, performance of a behaviour [34]. For example, some participants described engaging in lifestyle behaviours, or not, as consequence of their understanding of particular items of lifestyle information:

FM2 (male): Well, red wine reduces your cholesterol - well that has been my excuse for [a] long [time]! I took it to reduce cholesterol! That's why people in, like France and the Mediterranean countries, don't have anywhere near as many heart attacks as we do. Not just their diet, but they drink quite a lot of red wine. So I thought, 'Well, if the Spanish can do it, why not me?' (FG5)

And,

PwS12 (male): I thought, 'If I want to have a better quality of life, I [should] stop smoking!' (FG4)

In terms of whether or not people received information about lifestyle behaviours in relation to the secondary prevention of stroke, the majority of participants initially reported having received little or no information when they were in hospital. However, when this was explored further, some participants did describe having received general lifestyle information i.e. information that covered all the relevant lifestyle topics (i.e. alcohol, diet, physical activity, tobacco):

FM1 (male): When my wife left hospital, we got a pack of information. (FG1, not verbatim)

And,

PwS9 (female): Well, we got a book ['My stroke', which contains a range of information, including generic lifestyle information] from the ward. (FG3)

In terms of dietary information in particular, some participants reported having received specific lifestyle information, particularly if the person who had had the stroke had diabetes or dysphagia. With regards the accessibility of information, information was most commonly provided in written format i.e. leaflets, folders and books and was not reinforced verbally:

FM12 (male): I think there was a folder right at the beginning that I got but I think it had to be left in the hospital, you know, I don't think I got it home with me ... Yeah, as [FM16] said, just pamphlets and things like that but never any spoken words, or anything about it. (FG7)

However, as a few participants pointed out, the ability to read, concentrate and assimilate information may be adversely affected in the early stages of recovery:

PwS11 (male): ... a stroke affects people in different ways ... when I first got out of hospital I was finding reading irritating [general agreement], not impossible, just irritating. (FG4)

And,

PwS16 (male): ... when you've first had your stroke, you're in no condition to absorb anything. (FG6)

In addition, a few participants observed that information in written formats is inappropriate for people with aphasia and other perceptual or cognitive impairments, as the following quotes exemplify:

GP (communication support): You did get information, [uses participant's name]?

*PwA2 (female): Yes*.

*GP: ... Do you want to try and write it [GP takes some time with participant who tries to write something; there is a pause in the discussion]*.

GP: Right, let's have a look. Mmm, was it a leaflet ... did you get a leaflet ...?

PwA2 (female): Yes, yes, thank you. (FG3)

And,

*FM22 (male): We were given pamphlets about healthy eating; still got them*.

PwA7 (female): Oh, I can't remember that! (FG8)

The need to include family members was highlighted by those participants who had a relative with aphasia, and who had direct experience of the frustration caused by exclusion from the information giving process:

FM21 (female): I found the worst thing when my husband was in hospital was that he received a lot of information. The dietician visited him, the physio visited and gave him information, but he [had] lost the power of speech so he was never able to communicate anything of that to me. (FG7)

Some participants discussed the issue of providing secondary prevention information at an appropriate stage of the recovery process. They identified stages at which they felt that information-giving was inappropriate and/or ineffective, e.g. in the acute phase post-stroke, and stages when they felt it would be most beneficial, e.g. once they were settled back at home, following discharge from hospital:

ML: When would it be best to get this kind of information?

*FM10 (male): probably prior to discharge or after discharge ..*.

*PwS18 (female): I still don't think you can take it all in [over-talking]*.

*PwS16 (male): I think it depends on the severity of your stroke. I mean obviously everyone's had different strokes ..*.

*PwA6 (female): Uhhuh, uhhuh [sounds of agreement]*.

PwS16 (male): ... I think about three months [after discharge home]. (FG6)

And,

*FM14 (male): I think at the time the person takes the stroke, you are more concerned, you know, you possibly get things and you don't remember, whereas if ... the stroke nurse does all that [post-discharge], you seem to get more information ..*.

*FM18 (female): I think when the person is in hospital, I think all you're wanting to see is getting them out of hospital, I think that's your main concern, first their wellbeing, and then ..*.

FM12 (male): ... are they going to survive it, for a start?

FM18 (female): ... aye, and then [over-talking; general agreement 'that's it'] you want to get them home. (FG7)

However, the lack of clear consensus on this issue reinforced participants' sense that individual experiences of stroke and of recovery from stroke vary considerably.

### Behavioural beliefs

Behavioural beliefs influence attitudes towards a particular behaviour. A commonly held belief amongst participants was that knowledge about the components of healthy lifestyle behaviours was 'common knowledge' and a matter of 'common sense'. Many participants believed that they had a tacit understanding of what constitutes healthy lifestyle behaviour:

PwA3 (female): I think everybody really knows about drinking, and fatty foods and smoking. (FG4)

And,

FM4 (female): ... eating and drinking - it's common sense really. (FG5)

However, when this was explored further, it was evident that there was a general perception amongst participants that the healthy lifestyle information promoted in public health campaigns was subject to constant change, resulting in uncertainty regarding the nature of healthy lifestyle behaviour:

FM2 (male): Can I ask you, 'what is healthy eating?' Because every time you pick up a paper, something that was good for you yesterday is bad for you today! [laughter; general agreement] ... When somebody says to me healthy eating and healthy diet ... I react rather badly because ... today's healthy diet is tomorrow's, 'Don't eat that, it's going to kill you!' (FG5)

One family member offered his definition of what constituted a 'balanced diet', a phrase frequently used by participants to describe their eating habits. The complexity of this definition demonstrates the breadth and depth of knowledge required in order to eat healthily.

*FM22 (male): ... it's a case of trying to just get a balanced diet. [My wife] eats plenty of fruit, she always eats fruit, em, not so keen on some of the red meats, she doesn't like that. Eh, chicken, she's not a fish person, the only fish she'll eat is out the chippy [takeaway fish and chip restaurant]! [all laugh] Which is not very often, once a week, maybe. But she has a pretty balanced diet ... I would say what we ate prior to her stroke, was still a pretty balanced diet although myself I always tried to eat balanced. I go for chicken and I like fish, which are good for you ... I was always careful about what I eat, I don't stuff myself full of fatty foods and that. [My wife] did the same, em, I think our diet is quite good, you know. (FG8)*.

Whilst most participants were aware that drinking too much alcohol and smoking are detrimental to health, a few participants also described alcohol and tobacco as having stress-relieving properties. Family members in particular reported drinking alcohol and using tobacco as a means of relieving some of the stress associated with their caring role:

*FM12 (male): I think, eh, the pressure that I'm under as a carer, I don't think that I could stop smoking at this particular time*.

FM17 (male): ... the stresses, you know, are quite horrendous, as [FM3] says ... every opportunity you ... get, you just light up a cigarette and go outside. (FG7)

Participants' described beliefs about the benefits of adopting healthy lifestyle behaviours that were influenced by personal experience. For example, several participants reported that, although they or a family member had made positive adjustments to their lifestyle behaviour(s), such action had not prevented them from having a stroke, thus challenging their belief that certain lifestyle behaviours are beneficial to health:

FM21 (female): My husband neither smoked nor excessively drank and he took a stroke. (FG7)

And,

*PwS12 (male): I gave up smoking for 18 months, stopped drinking and went on a diet and I still had a stroke*.

PwA3 (female): I was a smoker but I had stopped before I took the stroke. (FG4)

When discussing making changes to lifestyle behaviour following stroke, some participants described deciding that no change was necessary. In terms of diet, for example, this was because they believed they had a healthy diet prior to stroke:

PwS14 (male): ... [the dietician] talked to my wife about what we should be eating, what the diet should be and, of course, we were virtually doing that anyway, so I should never have had a stroke [general laughter]. (FG6)

However, other participants were clearly not ready to contemplate making positive changes to their diet [35], irrespective of its apparently 'unhealthy' content and the information and advice received from dieticians:

PwA7 (female): [recalling her conversation with a dietician] No, I just, eh, well, you know, you had to use margarine and you had to use that, and I said, 'Well, don't write it down on the thing, because I will never, ever take margarine.' Sometimes I'll not take [butter] at all, but I said, 'If I'm going to use it, it will be butter!' Things like that. 'Don't eat a chocolate biscuit! Don't eat [pauses] ... I said, 'If I fancy one, I'm going to have it!' (FG8)

With regards physical activity, some family members who were also carers believed that taking action to maintain a good level of physical fitness was of vital importance as it enabled them to continue in their essential role as carer:

FM14 (male): ... as [FM13] said, what happens to [my wife]? I'm coming up to 80 ... but I've got to look after my wife ... and so ... I've got a treadmill and I do that ...[because] it keeps me fit. (FG7)

### Normative beliefs

Normative beliefs concern the individual's perception of the expectations of others in relation to specific behaviours. For example, if an individual perceives that a specific behaviour is approved by family members, friends or members of wider social and other networks, this will influence their beliefs, attitudes and ultimately intention to engage in that behaviour.

In this study, many participants described the influence of normative beliefs on their intention to engage in healthy lifestyle behaviour(s). For example, participants described beliefs and attitudes held by family members that positively influenced their own beliefs and behaviour in terms of smoking, drinking alcohol, diet and physical activity:

PwS6 (female): My son bought me that ... exercise machine ... My husband he uses it as well; that helps me. (FG3)

And,

PwS13 (male): ... [my wife] doesn't go out and buy fatty foods. We do eat quite a lot of fish, fruit and vegetables as well ... I think it was just something we knew we had to, we knew we had to change. (FG4)

However, the influence exerted by family members was not always in favour of healthy choices and behaviours:

FM12 (male): I did try [to quit] when [my wife] decided to stop smoking, we went to the smoking cessation classes ... she stopped smoking and just recently she's started back again, but she blames me, because I smoke. (FG7)

Similarly, participants described the influence of friends and members of other social networks on lifestyle behaviour. One man recalled how the social aspect of his leisure activity negatively influenced his levels of drinking, prior to his stroke:

PwS14 (male): Well ... I was a binge drinker! ... because I was a bowler and of course when you bowl, it was a dram [a small measure of whisky] every second end, and you could drink nearly a bottle of whisky in a day, and you'd be bowling all day, and ... well over half a bottle would be no problem. That was more or less every Saturday during the summer. (FG6)

Conversely, one woman described the positive influence of her social network. She and her fellow stroke support group members believed that exercising safely following stroke was important to their recovery and well-being. Therefore, the group was committed to a programme of fundraising in order to secure the professional support required to enable them to take part in a weekly exercise class:

PwA3 (female): Another thing that we ... rely on [as] ... group members, is that we've now got a weekly session with a physiotherapist who is neurologically trained ... so we have to pay as a, as a group, we have to raise money in order for us to get proper, em, em, she's a Pilates teacher, she takes a few Pilates classes ... it's through our own efforts to raise the money, enough to put into the group to pay for the physiotherapist. (FG4)

An example of the role of social networks in terms of influencing behaviour was observed in one of the focus groups. Two family members who believed it was important to keep fit and healthy in order to maintain their essential roles as carers, tried to positively influence another group member to think in a similar way with regards smoking:

FM14 (male): You know, I was just thinking about you [to FM12] I mean I used to be a smoker as well ... [do] you never worry about it, something happening to you through smoking? What's going to happen to your wife? You know what I mean?

*FM12 (male): [slowly] Aye*.

*FM14 (male): So that's the kind of thing that motivates me more than anything to look after myself*.

*FM18 (female): That's right*.

*FM14 (male): I just couldn't bear for [my wife] to go into a home or something like that ... none of us would ... I'm just saying that's what really motivates me to look after myself*.

FM16 (male): I mean ... I watch my intake of food ... [and] I do exercise ... I know it's going to help her in the long run, because I've got to lift her and things like that. (FG7)

In contrast, another family member in the same group reinforced the belief that, in spite of the adverse effects of smoking and excessive alcohol consumption on health, it is common for carers to drink alcohol and to smoke tobacco in order to counteract the stressful effects of their caring roles, and that this was therefore 'expected' and indeed 'permissible' behaviour:

FM17 (male): There is a leaflet at the [hospital] ... it categorically states that if you are a smoker when you are caring with somebody with a stroke you will tend to smoke more, if you are a drinker you will drink more, things like that, because the stresses, you know, are quite horrendous ... you know [that] today is not your day, [so] every opportunity you get you just light up a cigarette. (FG7)

### Control beliefs

Control beliefs describe an individual's beliefs regarding their own skills and abilities, and the opportunities and resources available to them that may support their engagement in a particular behaviour.

Many participants cited the effects of stroke as presenting barriers to engaging in certain lifestyle behaviours, such as diet and physical activity. For example, depression is a common consequence of stroke [36], and some participants described an association between depression and a lack of motivation to eat healthily or to engage in physical activity for exercise:

PwA3 (female): I know that when I am, when I go through my 'plus' stages [i.e. not feeling depressed] I can feel a difference. When I'm exercising I can feel a difference in my personal [sense of well-being], when I want to get up in the morning, whereas normally I would lie and lie and lie and sleep. And I notice a difference in my walking and everything. So I know that going to the gym and going to keep fit sessions is good for me personally. (FG4)

Several other participants described the negative impact of physical effects of stroke, such as hemiplegia, on their ability to engage in physical activities:

PwS11 (male): It's not just as much the energy ... it's the concentration levels I throw at it ... when I'm standing up now, [laughs] I'm having to think about it ... [and] it does take an awful lot out of you to walk! (FG4)

However, other participants did not believe that physical impairments should prevent them from adopting healthy lifestyles, and elected to eat healthily and engage in wheelchair-based exercise:

PwS18 (female): I don't think I over eat [and] ... at night, you know, I do them [my exercises] sitting, I try and do them, my hands, my legs ... (FG6)

Many participants described a lack of resources designed to support healthy lifestyle behaviours. For example, some family members complained that the Home Help service (a support service delivered by social work departments) did not facilitate healthy eating:

FM3 (female): ... the Home Helps, they're not going to prepare something that's fantastically healthy ... they're going to do something they can 'ding ding' in the microwave. (FG5)

Others described a lack of easy access to appropriate exercise facilities. However, participants who did have access to such resources found this facilitated their ability to join appropriate clubs and groups and to engage with the associated lifestyle behaviours such as healthy eating and physical exercise:

PwS7 (female): I actually joined a slimming club as I had to lose weight ... I'm still going ... I've lost about two and a half stone. (FG3)

And,

PwS8 (female): Recently I've started swimming ... [the physiotherapist] got me a group to join ... because ... I needed someone in the pool with me ... this is a disabled group and they are very, very good. (FG3)

## Discussion

Analysis of the focus group data was influenced by aspects of TPB, which enabled understanding of participants' beliefs, attitudes and knowledge in relation to secondary prevention lifestyle information.

Typically, participants reported having received little or no secondary prevention/lifestyle information following stroke, although further probing revealed that participants did receive information, most usually in the form of leaflets or information folders. It is likely that patients and their families forget much of what they are told during the acute phase of recovery from stroke, as initially survival and getting back home again are their overriding concerns [37]. This suggests that patients and their families are most likely to be receptive to secondary prevention information once they have returned home and have been discharged to the care of community-based HCPs.

When further exploration revealed that many participants did recall having received information, it was noted that information was often given to, or made available to patients and/or their families but that there was little or no verbal reinforcement or discussion. There is a wealth of evidence, which indicates that information-giving alone is insufficient to ensure understanding and assimilation of the information provided [38]. Good practice requires that when providing information HCPs should utilise strategies which actively involve patients and their families, and which include planned follow up to ensure clarification and reinforcement are provided, as needed by the patient and/or their family [38]. In this study, participants emphasised the need for HCPs to ensure that the timing of information provision is a good 'fit' with individual patients' and families' abilities and priorities. Given the variable recovery trajectory following stroke, our findings indicate that community-based HCPs have an important role to play in determining the optimum timing for information provision. In order for information provision to be effective, it should be viewed as a person-centred or a family-centred issue, as appropriate [39]. The need for a family-centred approach was highlighted as being of particular relevance for patients with aphasia and their families [28,40].

In terms of behavioural beliefs, many participants described making healthy lifestyle choices as a matter of 'common sense' and 'common knowledge'. However, further investigation revealed that knowledge was often based on information acquired through a variety of media, including television and posters, and that participants found these public health messages to be confusing and contradictory, causing some participants to reject them as lacking credibility. This finding is supported by Price et al who, following their focus group study of people with Type 2 diabetes, developed a brief lifestyle intervention which avoided making reference to government health policy in order to ensure that recipients believed the information to be credible and reliable [41]. Undoubtedly, the information required to enable people to make healthy lifestyle choices is complex. As illustrated in this study, detailed knowledge is required across a broad area, and again this indicates the need for community-based HCPs to ensure that patients and their families have access to appropriate, evidence-based information. Participants also described instances where experiential learning or the misunderstanding of information had negatively effected behavioural beliefs and attitudes. These findings demonstrate the important influence of behavioural beliefs on individuals' and families' intention to engage, or not, in a particular behaviour. The need is highlighted for HCPs to attempt to elicit underlying beliefs before delivering lifestyle risk factor information or implementing behavioural interventions, and correcting any misunderstandings.

Increasingly, health promotion interventions designed to address a range of health conditions are targeting families, or social peer groups. For example, MEND (Mind, Exercise, Nutrition ... do it!), an intensive community-based, family-centred childhood obesity intervention is achieving significant results across a wide range of outcome measures including body mass index and physical activity [42]. And, MyAction, a vascular prevention initiative that adopts a family-centred, community-based approach to the primary and secondary prevention of cardiovascular disease, has demonstrated significant improvement in some aspects of lifestyle behaviour, including dietary outcomes and physical activity, over the course of a year [43]. In this study, the important influence of normative beliefs on an individual's intention to engage, or not, in a particular behaviour was clearly demonstrated, as families were seen to exert a strong influence, in either a positive or a negative direction, on each others' lifestyle beliefs and behaviours. Similarly, the influence exerted by social groups was described by participants and observed by the researchers. Visser-Meily et al advocate the need for a family-centred approach to stroke rehabilitation in order to improve the effectiveness of rehabilitation processes and thus improve outcomes for patients and their families [44]. Similarly, our analysis of the findings from this focus group study suggests that a family-centred approach would enhance the effectiveness of secondary prevention lifestyle interventions. It is of note that, in this study, in comparison with families and peer groups, only rarely were HCPs perceived to exert an influence on the lifestyle beliefs and behaviours of participants. Again, this suggests the need for community-based HCPs to instigate active information provision strategies ensuring that patients and their families have access to reliable secondary prevention information from credible sources [38]. However, a recent survey, undertaken as part of our programme of secondary prevention lifestyle research, highlighted that stroke nurses do not always have the knowledge and skills required to deliver effective health promotion interventions [21]. This suggests a need for HCP training and education that addresses this issue.

This study demonstrated that individual's control beliefs are influenced by the individual's and/or the family's perceptions of the limiting nature of the effects of stroke. Some participants (people who had had a stroke and family members) described stroke as limiting the individual's ability to engage in healthy lifestyle behaviours, others made appropriate accommodations and found ways to engage in healthy behaviours. Some participants described a lack of appropriate infrastructure and accessible resources, e.g. a few participants identified institutional barriers to healthy living, such as Home Helps having insufficient time to prepare healthy food. HCPs need to be able to offer appropriate psychological support to families following stroke and, once the patient has been discharged back to primary care, to support families in innovative ways of thinking and resource utilisation.

Finally, one key finding related specifically to family members who were carers for a close relative, often their spouse. These carers engaged in behaviours that were influenced by their knowledge or understanding of the information available to them, their expectations of outcomes related to specific behaviours, and the beliefs of their social peers. These participants variously engaged in healthy behaviours (i.e. regular exercise, a healthy diet, no tobacco and minimal alcohol consumption) or unhealthy behaviours (i.e. smoking tobacco and drinking alcohol) in order to be able to continue in their demanding role as carers. Both groups wanted to continue in their caring role; however, as indicated, very different strategies were adopted. This finding suggests that there is a need for community-based HCPs to address the underlying beliefs and influences associated with the lifestyle choices of family carers, before attempting to address with them issues of primary prevention and behaviour change.

### Limitations

In all but one of the groups (FG6), participants were known to each other as members of a pre-existing support group. However, this pre-participation familiarity may have served to facilitate discussion and to diffuse tension between group members when differences of opinion were voiced. In terms of transferability to other populations, although we attempted to recruit widely, we managed to recruit only one participant from a black and minority ethnic group. It is also worth noting that members of support groups are likely to have had more information and support than those who do not join such groups.

## Conclusions

The use of the TPB as a lens through which to view the data from this focus group study with people who have had a stroke and their families, enabled our understanding of the importance of beliefs in relation to behaviour and intentions to engage, or not, in healthy lifestyle behaviours. It is clear that simply having access to lifestyle information, and having a general awareness about what constitutes a healthy lifestyle are not sufficient to motivate and enable people to change their behaviour, even after a life-threatening event. Timing of information provision, context and social environment are major influential factors, as is the credibility of information sources. In particular, the findings highlighted the powerful nature of the influence exerted by family members on patterns of lifestyle behaviour within the family context. HCPs need to be cognisant of these important psychological processes and influential factors and use appropriate theoretical approaches to inform the design and delivery of information strategies and other secondary prevention interventions. This will support patients and their families in making sustained positive changes to specific lifestyle behaviours.

## Competing interests

The authors report no competing interests. The authors alone are responsible for the content and writing of the paper.

## Authors' contributions

ML contributed to the design of the study, collected and analysed the data and led the writing of the paper. SK contributed to the design of the study, the data collection and data analysis, and contributed to the writing of the paper. HW contributed to the design of the study, the data analysis, and the writing of the paper. GP contributed to the design of participant information sheets and consent forms, the data collection, and the writing of the paper. GE contributed to the design of the study and the writing of the paper. All authors read and approved the final manuscript.

## Authors' information

ML: PhD, MSc, MA (hons), RGN. ML has a background in neurological rehabilitation nursing and in information science. As a researcher, she has worked on a variety of projects including an investigation of the stressors faced by the carers of adults who have had a stroke and the experience of stroke from the perspective of young adults and their families. Her current stroke research is concerned with the role of lifestyle factor modification in the secondary prevention of stroke. ML is also Depute Director of the Scottish Centre for Evidence Based Care of Older People, a collaborating centre of the Joanna Briggs Institute.

SK: PhD, MSc (Med. Sci.), BA, RN, HV. SK, who is a public health nurse, has a particular interest in the social, environmental and behavioural determinants of health. She has undertaken a number of projects in the stroke field, including an investigation of stress and coping in carers. Her current stroke research focuses on secondary prevention through lifestyle modification. Key areas of expertise/interest include the development and evaluation of smoking cessation interventions and brief alcohol interventions. SK is the current Chair of the Scottish Tobacco Control Alliance Research Group and she is a member of the Research & Evaluation Subgroup of the Ministerial Group on Tobacco Control. She is also a former member of the Executive Committee of the Nursing Council on Alcohol.

HW: PhD, MN, RGN, RMN, RNT, currently Visiting Professor in Health Sciences, University West, Sweden; formerly Professor of Nursing at Glasgow Caledonian University. HW's research and teaching relates to the role of the nurse in health promotion, in particular, to the prevention and/or reduction of alcohol-related problems. HW was a founding member of the Nursing Council on Alcohol, an organisation that aims to raise awareness among nurses throughout the UK of their role in helping to prevent and reduce alcohol-related harm. She was Chair from 2002-2005 and remains a member of its Executive Committee.

GP: BSc (Hons). GP is a Senior Speech and Language Therapist. She has made a considerable contribution to the development of training and education resources for stroke health professionals, including the Stroke Training and Awareness Resources (http://www.stroketraining.org), and the Stroke Core Competencies and Advancing modules. Her research interests include the secondary prevention of stroke, patient experiences of post-stroke dysarthria, and the resumption of driving with aphasia following stroke.

GE: MBChB, MD, MRCP (Glasg). GE is a Consultant Geriatrician working in Lanarkshire and honorary Senior Clinical Lecturer with the University of Glasgow. He has an interest in stroke. His research interests including the running of a randomised controlled trial of nurse led behaviour modification post stroke. He has authored a Cochrane review of stroke liaison workers.

## Pre-publication history

The pre-publication history for this paper can be accessed here:

http://www.biomedcentral.com/1471-2296/11/97/prepub

## Supplementary Material

Additional file 1 **Participant information sheet for people with aphasia**. Click here for file

Additional file 2 **Consent form for people with aphasia**. Click here for file

Additional file 3 **Semi-structured topic guide**. Click here for file

## References

[B1] Duhamel F Talbot N A constructivist evaluation of family systems nursing interventions with families experiencing cardiovascular and cerebrovascular illness J Fam Nurs 2004 10 1 12 32 10.1177/1074840703260906

[B2] Thompson H Ryan A A review of the psychosocial consequences of stroke and their impact on spousal relationships Br J Neurosci Nurs 2008 4 4 177 184

[B3] Galimanis A Mono M Arnold M Nedeltchev K Mattle H Lifestyle and stroke risk: a review Curr Opin Neurol 2009 22 60 68 10.1097/WCO.0b013e32831fda0e 19155763

[B4] Department of Health Reducing brain damage: faster access to better stroke care. [Executive summary] 2005 London: TSO

[B5] Mackay J Mansah GA The atlas of heart disease and stroke 2004 Geneva: World Health Organization

[B6] Redfern J McKevitt C Wolfe C Risk management after stroke: the limits of a patient-centred approach Health, Risk Soc 2006 8 2 123 141 10.1080/13698570600677266

[B7] Hankey G Spiesser J Hakimi Z Carita P Gabriel S Time frame and predictors of recovery from disability following recurrent ischemic stroke Neurol 2007 68 3 202 205 10.1212/01.wnl.0000250327.73031.54 17224574

[B8] Department of Health National service framework for older people 2001 London: TSO

[B9] Scottish Intercollegiate Guidelines Network (SIGN) SIGN 108: Management of patients with stroke or TIA: assessment, investigation, immediate management and secondary prevention 2008 Edinburgh: SIGN

[B10] Ovbiagele B Saver J Fredieu A Suzuki S McNair N Dandekar R Razinia T Kidwell C PROTECT A coordinated stroke treatment program to prevent recurrent thromboembolic events Neurol 2004 63 1217 1222 10.1212/01.wnl.0000140493.83607.f1 15477541

[B11] Youman P Wilson K Harraf F Lalit K The economic burden of stroke in the United Kingdom PharmacoEconomics 2003 21 43 50 10.2165/00019053-200321001-00005 12648034

[B12] Strong K Mathers C Bonita R Preventing stroke: saving lives around the world. Lancet Neurol 2007 6 182 187 10.1016/S1474-4422(07)70031-5 17239805

[B13] Stroke Association "Nobody told me ..." Highlighting the importance of information for stroke survivors when they leave hospital 2005 London: Stroke Association

[B14] Healthcare Commission Survey of patients 2005: Stroke 2005 London: Commission for Healthcare Audit and Inspection

[B15] Lawrence M Kerr S Watson H Jackson J Brownlee M A summary of the guidance relating to four lifestyle risk factors for recurrent stroke: tobacco use, alcohol consumption, diet and physical activity Br J Neurosci Nurs 2009 5 10 471 476

[B16] Lawrence M Kerr S McVey C A systematic review of the effectiveness of secondary prevention lifestyle interventions designed to change lifestyle behaviour following stroke. [protocol] 2009 http://www.joannabriggs.edu.au/10.11124/01938924-201109430-00001 27820540

[B17] Ellis G Rodger J McAlpine C Langhorne P The impact of stroke nurse specialist input on risk factor modification: a randomised controlled trial [research letter] Age Aging 2005 34 4 389 392 10.1093/ageing/afi075 15955759

[B18] Joubert J Reid C Barton D Cumming T McLean A Joubert L Barlow J Ames D Davis S Integrated care improves risk factor modification after stroke: initial results of the Integrated Care for the Reduction of Secondary Stroke Model J Neurol, Neurosurg Psychiatry 2009 80 279 284 10.1136/jnnp.2008.148122 19010943

[B19] Park EW Schult JK Tudiver F Campbell T Becker L Enhancing partner support to improve smoking cessation Cochrane Database Syst Rev 2004 3 CD002928 15266469 10.1002/14651858.CD002928.pub2

[B20] Medical Research Council Developing and evaluating complex interventions: new guidance http://www.mrc.ac.uk Accessed 2008 Dec 10

[B21] Lawrence M Kerr S Watson H Jackson J Brownlee M A survey of stroke nurses' knowledge and practice regarding four secondary prevention lifestyle issues: tobacco use, alcohol consumption, diet and physical activity Br J Neurosci Nurs 2009 5 11 518 523

[B22] Lawrence M Kerr S Whyte R Watson H The Development of a Theoretical Framework for a Complex Lifestyle Intervention [abstract] Int J Qual Methods 2009 8 3 S25

[B23] Kelly M The role of theory in qualitative health research Fam Pract 2010 27 285 290 10.1093/fampra/cmp077 19875746

[B24] Wright LM Leahey M Nurses and families 2005 4 Philadelphia: F.A. Davis Company

[B25] Ajzen I The theory of planned behaviour Org Behav Hum Dec Proc 1991 50 179 211 10.1016/0749-5978(91)90020-T

[B26] Barbour R Making sense of focus groups Med Educ 2005 39 742 750 10.1111/j.1365-2929.2005.02200.x 15960795

[B27] Baker R Hinton R Barbour RS, Kitzinger J Do focus groups facilitate meaningful participation in social research? Developing focus group research 1999 London: Sage 79 98

[B28] Nordehn G Meredith A Bye L A preliminary investigation of barriers to achieving patient-centred communication with patients who have stroke-related communication disorders Top Stroke Rehabilitation 2006 13 1 68 77 10.1310/5K2W-P6CD-EFDF-8HG4 16581632

[B29] Townend E Brady M McLauglan K A systematic evaluation of the adaptation of depression diagnostic methods for stroke survivors who have aphasia Stroke 2007 38 11 3076 3083 10.1161/STROKEAHA.107.484238 17932334

[B30] Brennan A Worrall L McKenna K The relationship between specific features of aphasia-friendly written material and comprehension of written material for people with aphasia: An exploratory study Aphasiology 2005 19 693 711 10.1080/02687030444000958

[B31] Cottrell S Davies A North Bristol Trust Stroke talk: a communication resource for hospital care 2006 London: Connect Press

[B32] Connect Including people with a communication disability in stroke research and consultation. A guide for researchers and service providers 2007 London: Connect

[B33] Pope C Ziebland S Mays N Analysing qualitative data BMJ 2000 320 114 116 10.1136/bmj.320.7227.114 10625273 PMC1117368

[B34] Ajzen I Theory of Planned Behaviour: Frequently asked questions http://www.people.umass.edu/aizen/faq.html Accessed 23 June 2010

[B35] Prochaska J DiClemente CC The transtheoretical approach: crossing traditional boundaries of therapy Home wood 1984 Illinois: Dow Jones-Irwin

[B36] Hackett M Anderson C Predictors of depression after stroke: a systematic review of observational studies Stroke 2005 36 2296 2301 10.1161/01.STR.0000183622.75135.a4 16179565

[B37] Lawrence M Patient-centred stroke care: young adults and their families PhD thesis 2009 Glasgow: Glasgow Caledonian University, School of Health

[B38] Smith J Forster A House A Knapp P Wright JJ Young J Information provision for stroke patients and their caregivers 2008 2 Cochrane Database Syst Rev CD0001919 10.1002/14651858.CD001919.pub2 18425877

[B39] Burton C Living with stroke: a phenomenological study JAN 2000 32 2 301 309 10.1046/j.1365-2648.2000.01477.x 10964176

[B40] Mason I Developing aphasia-friendly services for stroke survivors Nur Times 2006 102 323 32 33

[B41] Price H Dudley C Barrow B Kennedy I Griffin S Holman R Use of focus groups to develop methods to communicate cardiovascular disease risk and potential risk reduction to people with type 2 diabetes Fam Prac 2009 26 351 358 10.1093/fampra/cmp041 19546119

[B42] Sacher PM Kolotourou M Chadwick P Cole TJ Lawson M Lucas A Singhal A Randomized controlled trial of the MEND Program: a family-based community intervention for childhood obesity Obesity 2010 18 S2 S1 S7 20107463 10.1038/oby.2009.433

[B43] Wood DA Kotseva K Connolly S Jennings C Mead A Jones J Holden A De Bacquer D Collier T De Backer G Faergeman O Nurse-coordinated multidisciplinary, family-based cardiovascular disease prevention programme (EUROACTION) for patients with coronary heart disease and asymptomatic individuals at high risk of cardiovascular disease: a paired, cluster-randomised controlled trial The Lancet 2008 371 1999 2012 10.1016/S0140-6736(08)60868-5 18555911

[B44] Visser-Meily A Post M Gorter J Berklekom S van den Bos T Lindeman E Rehabilitation of stroke patients needs a family-centred approach Disabil Rehabil 2006 28 24 1557 1561 10.1080/09638280600648215 17178619

