# Supplementary material for: An exploration of lifestyle beliefs and lifestyle behaviour following stroke: findings from a focus group study of patients and family members
Source: BMC Fam Pract. 2010 Dec 8;11:97. doi: 10.1186/1471-2296-11-97 (PMC3018456; doi:10.1186/1471-2296-11-97)
Supplement: Additional file 1 — Participant information sheet for people with aphasia. [file 1471-2296-11-97-S1.DOC]

You are being invited to take part in a research study.


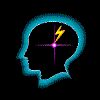


We want to find out what information you were given

**after your stroke** about:

Smoking
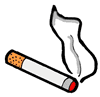


STROKE

Diet
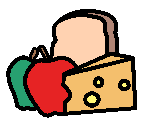


Exercise
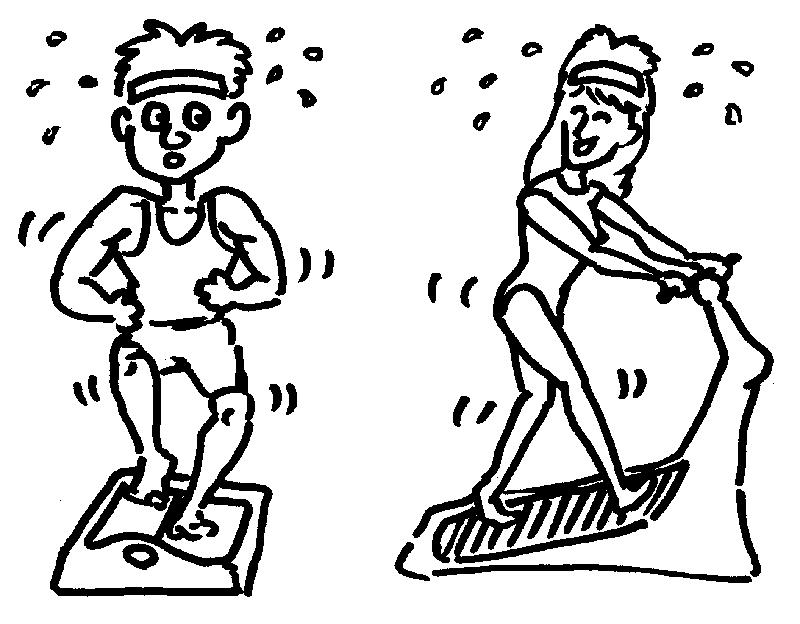


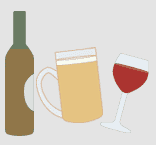


# Alcohol

This will help us to **improve** stroke services.

We would like you to come and talk to us about this.

It will be a group discussion with other people

who have had a stroke.


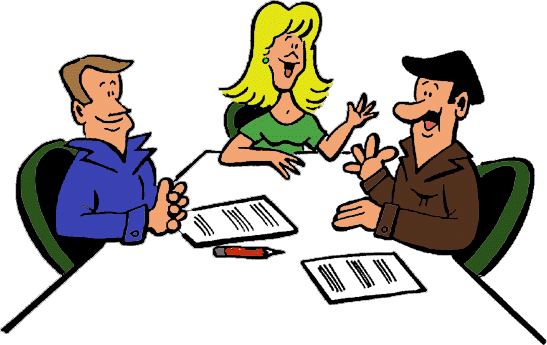


We will record the discussion.


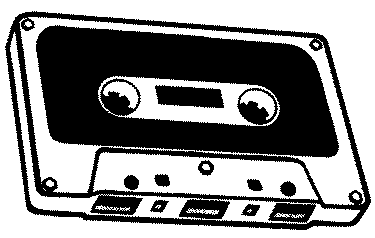


Everything you say is **private**. Your name will not be used.


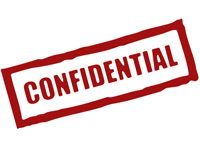


The recording will be destroyed at the end of the research.

You can stop at any time if you change your mind.

You can bring a friend or family member with you.


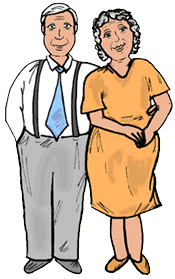


We will pay your travel expenses.

[
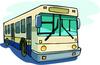
](http://www.barrysclipart.com/barrysclipart.com/showphoto.php?photo=20493&papass=&sort=1&thecat=998)
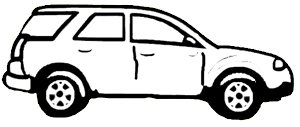

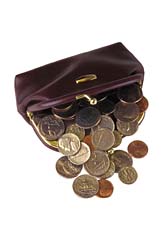


Would you like to be involved?

# **Yes**
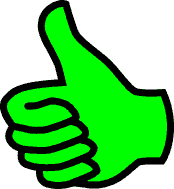


## OR


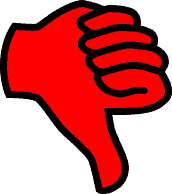


**No thanks**

If **Yes**,
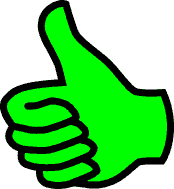


# Please sign the consent form. Send it to us in the envelope provided.


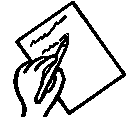

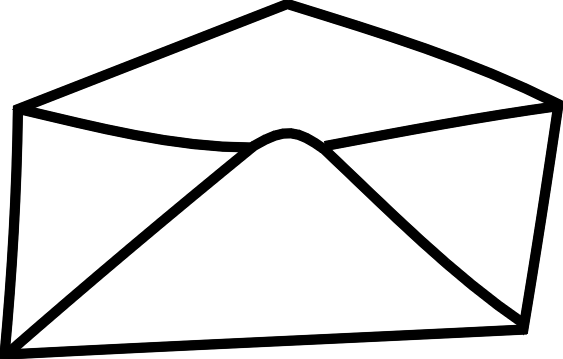


I will contact you and tell you more about the research.

*
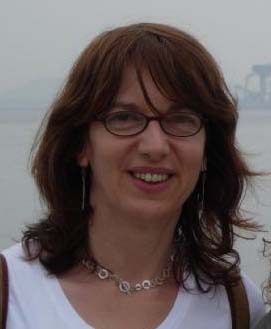
*

Maggie Lawrence

You can ask questions.

**You can contact:**

Maggie Lawrence

4^th^ floor, Buchanan House

Glasgow Caledonian University

Glasgow, G4 0BA


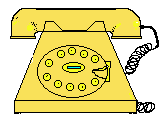
 0141 331 8863

**
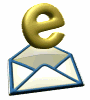
** margaret.lawrence@gcal.ac.uk
