# Supplementary material for: An exploration of lifestyle beliefs and lifestyle behaviour following stroke: findings from a focus group study of patients and family members
Source: BMC Fam Pract. 2010 Dec 8;11:97. doi: 10.1186/1471-2296-11-97 (PMC3018456; doi:10.1186/1471-2296-11-97)
Supplement: Additional file 2 — Consent form for people with aphasia. [file 1471-2296-11-97-S2.DOC]

| **CONSENT FORM** | | |  |
| --- | --- | --- | --- |
| **Healthy Living after stroke:**  **listening to what patients and their families say** | | |  |
| **We sent you information. It tells you about the research.** | | |  |
| 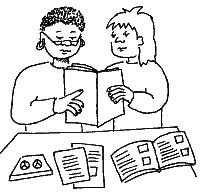 | - **Have you read the information?**   **YES / NO** | |  |
| 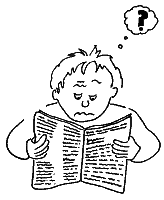 | - **Did you understand the information?**  YES / NO  - **Do you understand what will happen if you take part?**   **YES / NO** | | |
| **You can leave at any time. You do not have to say why.** | |  | |

| 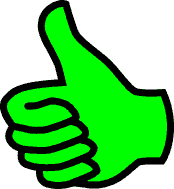 | - **Do you agree to take part?**   **YES / NO** |
| --- | --- |
| **Please complete the following:**  PRINT YOUR NAME: ____________________________  Date: ________________________________________  SIGN YOUR NAME: _____________________________  PRINT YOUR ADDRESS: _________________________  ____________________________________________  PHONE NUMBER: ______________________________ | |
| **Thank you for filling in this form.** | |
| For office use only:  Researcher: MAGGIE LAWRENCE  Researcher’s Signature:___________________________________ Date:_____________ | |
