# Supplementary material for: An exploration of lifestyle beliefs and lifestyle behaviour following stroke: findings from a focus group study of patients and family members
Source: BMC Fam Pract. 2010 Dec 8;11:97. doi: 10.1186/1471-2296-11-97 (PMC3018456; doi:10.1186/1471-2296-11-97)
Supplement: Additional file 3 — Semi-structured topic guide. [file 1471-2296-11-97-S3.DOC]

**TOPIC GUIDE**

During each focus group, the views will be sought of people who have had a stroke and family members of people who have had a stroke. Some focus groups will include people with aphasia and/or other communication impairments. Their communication support needs will be ascertained prior to the group meeting and the appropriate support provided e.g. large print documents or talking mats. The areas explored by the group will include:

- Knowledge of stroke risk factors (including lifestyle issues)
- Lifestyle information received (origin, type, mode of delivery)
- Perceived usefulness of any lifestyle information received (focus on tobacco, alcohol, diet & exercise)
- Perceived impact of any information received on lifestyle behaviours (focus on tobacco, alcohol, diet & exercise)
- Maintenance of behaviour change
- Knowledge of and access to services which support lifestyle behaviour change

There are 3 questions:

**Can you tell us what information you were given about healthy living after your stroke?** [information received]

**If you received information, did you find the information useful?** [utility; knowledge of risk factors]

**What did/didn’t help you to make lifestyle changes?** [attitudes and beliefs; facilitating factors/resources including the relevance and utility of any information received; support/services]

Topic guide

The purpose of today’s focus group is to find out what information people receive about healthy living after they have had a stroke (smoking, alcohol, exercise and diet). We would like to hear your views on any information you received and whether you thought it was useful.

But first of all, let’s start by going round the table introducing ourselves. And perhaps you would like to say why you decided to take part in this focus group today. I’ll go first ….

OK. Thank you for that, that was very interesting.

**Q.1** Let’s start the discussion by talking about healthy living, by that we mean smoking, alcohol, diet and exercise. **Please tell us what information you were given about healthy living after your stroke.**

[Use cards for the four subject areas to keep the discussion on track]

Prompts: [Who gave you the information?]

Perhaps you could describe what lifestyle information you received when you were in hospital? Who gave you the information?

What about when you got home? Your GP? Other community staff?

[Who was the information given to?]

Who did the [information giver] speak to? You alone? A member of your family? You and your family? You in a group with other patients?

[Format] Can you describe the type of information you received? e.g. Leaflets, large print, DVDs, etc

[Content] How well did the information meet your needs? Your family’s needs? e.g. general lifestyle information? Tailored lifestyle information?

[No information] What information would you have liked?

**Q.2** OK. I think now we’ve got a good idea about the lifestyle information you received [didn’t receive]. Perhaps now you could tell us **whether you thought that information was useful …**

[Use cards for the four subject areas to keep the discussion on track]

Prompts:

[Behaviour change]

What changes did you want to make to your lifestyle after you received the information?

What did you think/do after the Stroke Nurse gave you the leaflets?

What did you think/do after the GP spoke to you about stopping smoking/changing your diet?

[Involving family members]

Who did you talk to about the information? Someone in your family?

What did your family want to do after they got/you showed them the information?

**Q.3** OK. Well, we’ve talked a little about whether or not the information you’ve received has been useful. Some of you have mentioned that you wanted to make lifestyle changes after your stroke. **Can you describe what helped you to make those lifestyle changes?**

[Use cards for the four subject areas to keep the discussion on track]

Prompts:

[Family]

Can you describe how did your family helped? E.g. started eating healthy food; stopped smoking too

[Resources]

Can you describe what was available to help you?

Availability: e.g. classes, information (e.g. labelling on food and alcohol)

Accessibility: transport to attend classes, access to buildings, classes which will include people who have had a stroke

Cost: classes, transport, food etc

Convenience: e.g. I’ve started exercising because it fits in with my work …

[Support]

Can you describe who was available to help you?

One-to-one support; attending a group;

[Maintenance of change]

How easy was it for you to keep up the change(s) to your lifestyle?

**Q. 3a** **What didn’t help?**

Prompts: For example, why do the smokers amongst us keep smoking?

What stops us from ‘keeping fit’?

Q.4 In an ideal world, what information and support would you like? [people; resources, etc]

[Use cards for the four subject areas to keep the discussion on track]

OK. Well this has been a really interesting and useful discussion and I’m sorry to have to draw it to a close.

I am just going to take a minute to check that I haven’t missed. Does anyone have anything they would like to add, that we haven’t covered?

OK, thank you.

If you do think of something you would like to say at a later date, please get in touch. My contact details are on the information sheet or you can contact me through [*Group Leader’s name*].

Before we finish I’d like to thank [*Group Leader’s name*] for helping us to organise this discussion and a big thank you to all of you for helping us with this research. We will let you know the results – I’ll send copies to [*Group Leader’s name*].

We have travel expense forms here so that we can reimburse you for your travel expenses today.

[Refreshments are available…]
